# Effect of Controlled Artificial Disorder on the Magnetic Properties of EuFe_2_(As_1−*x*_P_*x*_)_2_ Ferromagnetic Superconductor

**DOI:** 10.3390/ma14123267

**Published:** 2021-06-13

**Authors:** Sunil Ghimire, Marcin Kończykowski, Kyuil Cho, Makariy A. Tanatar, Daniele Torsello, Ivan S. Veshchunov, Tsuyoshi Tamegai, Gianluca Ghigo, Ruslan Prozorov

**Affiliations:** 1 Ames Laboratory, Ames, IA 50011, USA; ghimire@iastate.edu (S.G.); kcho@ameslab.gov (K.C.); tanatar@ameslab.gov (M.A.T.); 2 Department of Physics & Astronomy, Iowa State University, Ames, IA 50011, USA; 3 Laboratoire des Solides Irradiés, CEA/DRF/lRAMIS, École Polytechnique, CNRS, Institut Polytechnique de Paris, F-91128 Palaiseau, France; marcin.konczykowski@polytechnique.edu; 4 Politecnico di Torino, Department of Applied Science and Technology, 10129 Torino, Italy; daniele.torsello@polito.it (D.T.); gianluca.ghigo@polito.it (G.G.); 5 Istituto Nazionale di Fisica Nucleare, Sezione di Torino, 10125 Torino, Italy; 6 Department of Applied Physics, The University of Tokyo, Hongo, Bunkyo-ku, Tokyo 113-8656, Japan; veshchunov@ap.t.u-tokyo.ac.jp (I.S.V.); tamegai@ap.t.u-tokyo.ac.jp (T.T.)

**Keywords:** tunnel diode resonator (TDR), coplanar waveguide resonator (CPWR), iron-based superconductors (IBS)

## Abstract

Static (DC) and dynamic (AC, at 14 MHz and 8 GHz) magnetic susceptibilities of single crystals of a ferromagnetic superconductor, ${\mathrm{EuFe}}_{2}{\left({\mathrm{As}}_{1-x}P_{x}\right)}_{2}$ (*x* = 0.23), were measured in pristine state and after different doses of 2.5 MeV electron or 3.5 MeV proton irradiation. The superconducting transition temperature, $T_{c}\left(H\right)$, shows an extraordinarily large decrease. It starts at $T_{c}\left(H=0\right)\approx 24\;K$ in the pristine sample for both AC and DC measurements, but moves to almost half of that value after moderate irradiation dose. Remarkably, after the irradiation not only $T_{c}$ moves significantly below the FM transition, its values differ drastically for measurements at different frequencies, ≈16 K in AC measurements and ≈12 K in a DC regime. We attribute such a large difference in $T_{c}$ to the appearance of the spontaneous internal magnetic field below the FM transition, so that the superconductivity develops directly into the mixed spontaneous vortex-antivortex state where the onset of diamagnetism is known to be frequency-dependent. We also examined the response to the applied DC magnetic fields and studied the annealing of irradiated samples, which almost completely restores the superconducting transition. Overall, our results suggest that in ${\mathrm{EuFe}}_{2}{\left({\mathrm{As}}_{1-x}P_{x}\right)}_{2}$ superconductivity is affected by local-moment ferromagnetism mostly via the spontaneous internal magnetic fields induced by the FM subsystem. Another mechanism is revealed upon irradiation where magnetic defects created in ordered ${\mathrm{Eu}}^{2+}$ lattice act as efficient pairbreakers leading to a significant $T_{c}$ reduction upon irradiation compared to other 122 compounds. On the other hand, the exchange interactions seem to be weakly screened by the superconducting phase leading to a modest increase of $T_{m}$ (less than 1 K) after the irradiation drives $T_{c}$ to below $T_{m}$. Our results suggest that FM and SC phases coexist microscopically in the same volume.

## 1. Introduction

Coexistence and competition of superconductivity and magnetism is a fascinating and actively studied topic. It is impossible to give even remotely-complete reference list, see for example Refs. [1,2,3,4,5,6,7,8,9,10]. Full local-moment ferromagnetism can destroy superconductivity even when it forms well below the superconducting transition temperature, $T_{c}$, for example in ${\mathrm{ErRh}}_{4}B_{4}$. However, even in this case, there is a narrow, but rich regime of the microscopic coexistence of two quantum phases [8,11,12,13,14]. Itinerant ferromagnetism may also coexist with superconductivity and such materials exhibit some very unusual properties [15]. Most of the studied ferromagnetic superconductors are singular compositions, which somewhat limits the possibility to study the trends and variations of properties in the continuous phase space, such as temperature vs. doping, $T\left(x\right)$ [7,8,10]. In contrast, there are many antiferromagnetic (AFM) superconductors where the regime of coexistence is easier to realize. Superconductivity develops on an AFM background as long as the internal magnetic field modulation occurs at distances much shorter than the superconductor’s coherence length, ξ, which is often realized in real materials. In turn, antiferromagnetism is largely unaffected by superconductivity because screening of the magnetic field is effective on the length scale of London penetration depth, $\lambda_{L}$ [3,6]. However, if the FM state is formed via the RKKY exchange interaction, superconducting pairing of conduction electrons may also affect the strength of the ferromagnetic exchange. In general, some form of spin arrangement with a net ferromagnetic component can be realized in a broad range of compositions in several families of magnetic superconductors, such as borocarbides [16,17,18] and more recently in some iron-based superconductors (IBS), where a decade of intense studies have clearly shown that magnetism plays an important, if not pivotal, role in their physics [19,20,21,22,23,24,25,26]. In the majority of IBS magnetism arises from the iron sublattice with spins aligned in the Fe-As plane where superconducting condensate mostly resides. However, in a few IBS compounds, there is an additional magnetism coming from, for example, europium as part of their formula [25]. In ${\mathrm{EuFe}}_{2}{\mathrm{As}}_{2}$, Eu${}^{2+}$ ions ($7\mu_{B}$ full local magnetic moment) order in an A-type antiferromagnet below 19 K while the iron sublattice develops a spin-density-wave (SDW) below 190 K [27,28]. The effect of ${\mathrm{Eu}}^{2+}$ magnetism is so large that can even be used to detwin the material by applying an in-plane magnetic field [29]. Thanks to a possibility of a continuous doping of the parent compound, superconductivity can be induced in some range of compositions by isovalent substitution of phosphorus for arsenic. With increasing *x* in ${\mathrm{EuFe}}_{2}{\left({\mathrm{As}}_{1-x}P_{x}\right)}_{2}$, the Eu${}^{2+}$ spins become canted out of the ab−plane producing a net ferromagnetic component along the *c*-axis. In our crystals with x=0.23, in zero applied magnetic field, superconducting transition occurs upon cooling at $T_{c}\left(H=0\right)\approx 24\;K$, followed by the magnetic transition of europium sublattice at $T_{m}\approx$ 18 K [25]. Although rare, this is not a singular FM/SC composition in this IBS family. In a related compound, ${\mathrm{RbEuFe}}_{4}{\mathrm{As}}_{4}$, ferromagnetism develops at $T_{m}\approx 15\;K$ in a superconducting background with $T_{c}\left(H=0\right)\approx 36.5\;K$ [30,31]. It is worth noting that while most theories address the coexistence of magnetism and superconductivity in IBS with respect to the iron ions [22,26,32], only few specifically target magnetism coming from other ions, such as ${\mathrm{Eu}}^{2+}$ [33,34,35].

When studying complex non-stoichiometric materials it is important to be able to fix the composition and examine the evolution of field and temperature dependencies when some other non-thermal control parameter is varied. One obvious example of such parameter is pressure, which has been used intensely for this purpose. Another is a controlled disorder that provides an important insight into magnetism and superconductivity [36,37,38,39,40,41,42,43,44,45,46,47], in particular in IBS [24,44,45,48,49,50,51,52,53]. Additional scattering can be induced by various means ranging from chemical substitution [54] to particle irradiation [55]. Controlled disorder has been used to study superconductors since the times of the famous Anderson theorem [37] and Abrikosov-Gor’kov theory [38], where the main attention was paid to the variation of the superconducting order parameter, hence the experimentally accessible transition temperature, $T_{c}$ [2,7,9,46,56,57,58]. More recently, the response to the variation of the scattering rate was studied for other properties, such as superfluid density and thermal conductivity, which are directly linked to the superconducting order parameter structure [40,44,45,49,51,59,60,61]. Due to relative rarity of magnetic superconductors, there is limited experimental information on the effects of disorder simultaneously on the superconductivity and magnetism. While we are not aware of such studies in magnetic borocarbides, in IBS the effect of disorder on magnetism and superconductivity was studied in several works [62,63,64,65], but none of them on the ferromagnetic Eu-based 122 compounds, except for a recent study of the effect of proton irradiation on the subject compound by some of the authors [66].

In the present work, we study the effects of electron irradiation on single crystals of ${\mathrm{EuFe}}_{2}{\left({\mathrm{As}}_{1-x}P_{x}\right)}_{2}$ and compare with the proton irradiation performed on similar samples. We find that in this particular ferromagnetic superconductor, magnetic and superconducting subsystems coexist almost independently. Superconductivity interacts with the internal magnetic field produced by the Eu${}^{2+}$ sublattice and the ferromagnetism is barely screened by the superconducting phase. The non-trivial interaction is revealed when the artificial point-like disorder enhances both potential and spin-flip scattering channels affecting $T_{c}$ at a much greater rate compared to the nonmagnetic IBS. Remarkably, controlled disorder combined with almost reversible annealing allows examining the properties of both phases in the regimes of $T_{c}>T_{m}$ and $T_{c}<T_{m}$ in a single composition.

## 2. Experimental

**Crystal growth, samples**. Single crystals of ${\mathrm{EuFe}}_{2}{\left({\mathrm{As}}_{1-x}P_{x}\right)}_{2}$, *x* = 0.23, were grown using self flux method from Eu powder (3 N purity), FeAs and FeP precursors, mixed stoichiometrically with nominal *x* = 0.25 [67]. The batch was grown inside stainless steel tube in nitrogen atmosphere with $T_{max}=1350{\;}^{\circ }$C (heating at a rate of 50 ${}^{\circ }$C/hour, keeping at $T_{max}$ for 12 h) followed by slow cooling at 2 ${}^{\circ }$C/hour down to $T_{min}=1000{\;}^{\circ }$C.

**Tunnel Diode Resonator (TDR)**. The real part of the radio-frequency magnetic susceptibility was measured by using a sensitive tunnel diode resonator (TDR) [68,69,70]. The sample (typically $∼0.5\times 0.5\times 0.1\;{\mathrm{mm}}^{3}$) is mounted on a sapphire rod using a trace amount of Apiezon^TM^ N-grease in a desired orientation and inserted into the inductor coil. The coil generates a small AC excitation magnetic field, $H_{ac}\approx 1-10\;A/m$, the exact value of which depends on the distance between the coil and a copper tube in which the coil is housed for temperature stability and electromagnetic shielding. The other end of the sapphire rod is glued into a copper block containing a Cernox^TM^ thermometer and a resistive heater.

In the experiment, the resonant frequency of the LC tank circuit with the sample inside the coil is recorded as function of temperature or external DC magnetic field, generated by the superconducting magnet outside the cryostat. The shift of the resonant frequency, $\Delta f=f\left(H,T\right)-f_{0}$, from its value without the sample, $f_{0}$, is proportional to the sample magnetic susceptibility [68,71]:

(1) $$\Delta f\equiv f\left(H,T\right)-f_{0}=-\frac{f_{0}V_{s}}{2V_{c}}\chi \left(H,T\right)$$

where χ(H,T)=dM/dH is the actual magnetic susceptibility of a given sample with volume magnetization $M=m/V_{s}$, where *m* is total measured magnetic moment. In paramagnetic samples χ>1, then the total inductance of the sample inside the coil increases, and resonant frequency decreases, whereas in a diamagnetic sample the opposite is true. In a superconducting sample, the magnetic susceptibility of a superconductor is given approximately by [68,71]

(2) $$\left(1-N\right)\chi \left(H,T\right)\approx \frac{\lambda }{R}\tanh\frac{R}{\lambda }-1$$

where *N* is the effective demagnetizing factor [72] and *R* is the effective dimension calculated numerically for a particular sample geometry [68]. Considering a superconducting sample with magnetic penetration depth λ≪R, where 2R is the size of the sample in the direction of magnetic field penetration (field penetrates from two sides), we obtain for the penetration depth:

(3) $$\Delta \lambda \equiv \lambda \left(H,T\right)-\lambda \left(0,0\right)\approx R\frac{2V_{c}\left(1-N\right)}{f_{0}V_{s}}\Delta f=G\Delta f$$

where *G* is the calibration constant. The main source of uncertainty in *G* is the approximate factor

(4) $$\Delta f_{0}=\frac{f_{0}V_{s}}{2V_{c}\left(1-N\right)}$$

which gives the change in frequency when an ideal diamagnetic sample of the same shape and volume as the sample under study is inserted at base (theoretically at zero) temperature into the coil. The approximate Equation (4) is based on an idealized picture of an infinite solenoid where the sample perturbs the magnetic flux inside. For a realistic finite coil, Equation (4) is only a rough approximation. However, $\Delta f_{0}$ can be measured directly by mechanically pulling the sample out of the coil at the base temperature. Our cryostat is equipped to do just that, so we determine $\Delta f_{0}$ directly for each sample. Then,

(5) $$G=\frac{R}{\Delta f_{0}}$$

which shows the simplified meaning of constant *G* as the frequency shift when magnetic field penetrates the entire sample (of size 2R, from two sides, travelling distance *R* from each side).

The measurement was conducted down to 400 mK using Janis wet Helium-3 cryostat and a DC magnetic field that can be provided by the superconducting magnet ranges up to 9 T. Further details and applications of TDR technique can be found elsewhere [68,69,70,71].

**Coplanar Waveguide Resoantor (CPWR)**. The coplanar-waveguide-resonator technique allows determination of the complex permeability of small samples coupled to the resonator, within a cavity perturbation approach [73,74]. The presence of the sample coupled to the resonator induces changes in the resonance frequency and quality factor of the CPWR, that are related to the real and imaginary parts of the total AC susceptibility, respectively [66]:

(6) $$\begin{array}{cc} ℜ\chi & \approx 1-\frac{2\Delta f/f_{0}}{\Gamma_{f}}\\ ℑ\chi & \approx \frac{\Delta \left(1/Q\right)}{\Gamma_{Q}} \end{array}$$

where $\Delta f/f_{0}$ and Δ(1/Q) are the experimental shifts of the resonance frequency and of the inverse of the quality factor induced by the presence of the sample under test, and $\Gamma_{f}$ and $\Gamma_{Q}$ are geometrical factors that can be determined by a self-consistent procedure, which takes into account also the finite size of the crystal and the consequent demagnetization effects [75]. The overall real and imaginary parts of the susceptibility for ferromagnetic superconductors are given by a bulk magnetic contribution and by a screening given by the superconducting condensate. The superconducting transition temperature $T_{c}$ corresponds to the onset of the diamagnetic signal (onset of an increase of the resonance frequency upon cooling), while the magnetic transition temperature $T_{m}$ is defined as the onset of a positive contribution to the bulk susceptibility [76].

**Electron irradiation.** The 2.5 MeV electron irradiation was performed at the SIRIUS Pelletron linear accelerator facility operated by the Laboratoire des Solidés Irradiés (LSI) at the Ecole Polytechnique in Palaiseau, France. At 2.5 MeV electrons are moving with relativistic speed of 0.985*c* and the total flux of electrons is about 2.7 A of electric current through a 5 mm diameter diaphragm. The acquired irradiation dose is measured by a calibrated Faraday trap behind the sample and is conveniently expressed in $C\cdot {\mathrm{cm}}^{-2}$, where 1 $C\cdot \mathrm{cm}{}^{-2}=6.24\times 10^{18}$ electrons/cm${}^{2}$. Electrons are particularly useful, because unlike heavier particles, they produce well-separated point like defects, called Frenkel pairs (vacancy+interstitial). But even with electrons, the irradiation needs to be conducted at low temperature, in liquid hydrogen in our case to prevent rapid clusterization of newly formed Frenkel pairs. Upon warming up the interstitials leave the system via various sinks, such as surfaces, defects, dislocations etc and a metastable population of vacancies remains. Their concentration is determined by the highest temperature reached—we re-checked the irradiated samples after a year on the shelf at room temperature with no noticeable change. We describe the annealing experiment in the main text. In 122 IBS, we estimate that warming up from 22 K of irradiation run to the room temperature, about 70% of induced scattering centers survives as determined from in-situ resitivity measurements [77,78].

## 3. Results

**Figure 1:**
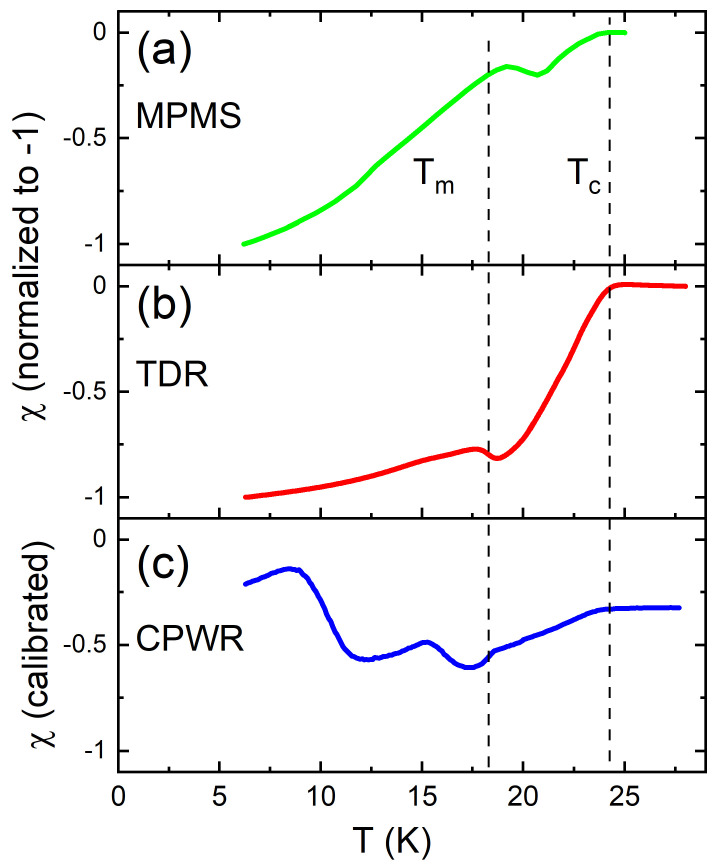
Temperature-dependent DC and real part of AC magnetic susceptibility of pristine EuFe${}_{2}$(As${}_{0.77}$P${}_{0.23}$)${}_{2}$ single crystals measured at very different frequencies: (**a**) SQUID magnetometer (MPMS, Quantum Design, DC regime, $H_{DC}=5\;\mathrm{Oe}$); (**b**) tunnel-diode resonator (TDR, 14 MHz, $H_{AC}=20\;\mathrm{mOe}$), and (**c**) coplanar waveguide resonator (CPWR, 8 GHz, $H_{AC}\approx 1\;\mathrm{Oe}$). Magnetic susceptibility is normalized to −1 at low temperatures for (**a**,**b**), but is shown in absolute values in a calibrated experiment in panel (**c**).

Figure 1 shows the temperature-dependent dynamic susceptibility of single crystal ${\mathrm{EuFe}}_{2}{\left({\mathrm{As}}_{0.77}P_{0.23}\right)}_{2}$ in pristine state measured using experimental techniques with very different time windows. Panel (a) shows the DC results obtained by using Quantum Design magnetic property measurement system (MPMS) at $H_{DC}=5\;\mathrm{Oe}$, panel (b) shows 14 MHz tunnel-diode resonator (TDR) data with excitation AC field of $H_{AC}=20\;\mathrm{mOe}$, and panel (c) shows 8 GHz data at $H_{AC}\approx 1\;\mathrm{Oe}$ obtained by coplanar waveguide resonator (CPWR) technique.

For comparison, the data in panels (a) and (b) were normalized to extrapolate to χ=−1 at the lowest T, whereas panel (c) shows the calibrated data. All three susceptibility curves clearly show superconducting transition near $T_{c}\approx 24\;K$ and ferromagnetic transition at $T_{m}\approx$ 18 K. The microwave-frequency CPWR data show extra features and a detailed analysis of the measurements is given elsewhere [66,76], while we are interested in a comparison of the transition temperatures. Below $T_{c}$ diamagnetic susceptibility is rather broad compared to much sharper transitions of nonmagnetic superconductors. This can be attributed to a substantial pairbreaking coming from the large-moment paramagnetic background. In the simple picture, if $\mu \left(T\right)$ is the normal state magnetic permeability, then the measured magnetic susceptibility is renormalized as [79,80]:

(7) $$\left(1-N\right)\chi \left(T\right)=\frac{\sqrt{\mu \left(T\right)}\lambda_{L}\left(T\right)}{R}\tanh\frac{\sqrt{\mu \left(T\right)}R}{\lambda_{L}\left(T\right)}-1$$

where *N* is the effective demagnetizing factor, *R* is the effective dimension and $\lambda_{L}\left(T\right)$ is the London penetration depth without magnetism present. Below $T_{c}$ the $\sqrt{\mu \left(T\right)}\lambda_{L}\left(T\right)$ term dominates the behavior with two competing trends. Taking the simplest functional forms, in the interval $T_{m}<T<T_{c}$,

(8) $$\sqrt{\mu \left(T\right)}\lambda_{L}\left(T\right)∼{\left(\left(T-T_{m}\right)\left(T_{c}-T\right)\right)}^{-1/2}$$

which is, indeed, a non-monotonic function of temperature in this interval which is seen in all three measurements shown in Figure 1. Below $T_{m}$ the magnetic susceptibility decreases and the overall signal tends to decrease again. Of course, the physics around ferromagnetic transition is significantly affected by the proliferation of spontaneous (vortex-antivortex) phases as was determined in the comprehensive microscopic study [81]. This scenario has been further explored in [76,82] Similarly, effects of spontaneous vortex phase was investigated both experimentally and theoretically in already mentioned 1144 sibling compound, ${\mathrm{RbEuFe}}_{4}{\mathrm{As}}_{4}$ [30,31,83,84].

**Figure 2:**
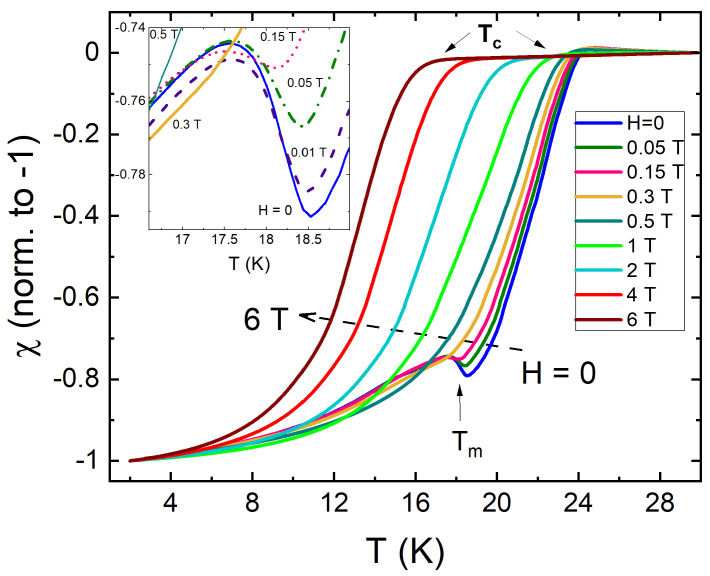
Normalized magnetic susceptibility of pristine EuFe${}_{2}$(As${}_{1-0.23}$P${}_{0.23}$)${}_{2}$ single crystal from TDR measurements at different DC magnetic fields applied along the *c*-axis. The inset zooms at the rapid suppression of the peak near the ferromagnetic transition.

Figure 2 shows TDR measurements of temperature dependent magnetic susceptibility of pristine ${\mathrm{EuFe}}_{2}{\left({\mathrm{As}}_{0.77}P_{0.23}\right)}_{2}$ at different magnetic fields applied along the *c*-axis. As expected, $T_{c}\left(H\right)$ decreases with the increasing magnetic field while $T_{m}$ remains practically unchanged. However, as shown in the inset in Figure 2, at the same time the height of the peak near $T_{m}$ decreases and disappears completely above 0.2 T. This is a characteristic behavior associated with a local moment ferromagnetism as shown previously using TDR technique [85].

### 3.1. Electron Irradiation

We now turn to the effects of the artificial disorder induced by the 2.5 MeV electron irradiation. Details of the experiment are described in Section 2. The irradiation dose is measured during the experiment as a total charge flux passed through the sample and can be expressed in convenient practical units of coulomb per cm${}^{2}$ to represent the irradiation dose, $1\;{C/\mathrm{cm}}^{2}=6.24\times 10^{18}\;\mathrm{electrons}/{\mathrm{cm}}^{2}$.

**Figure 3:**
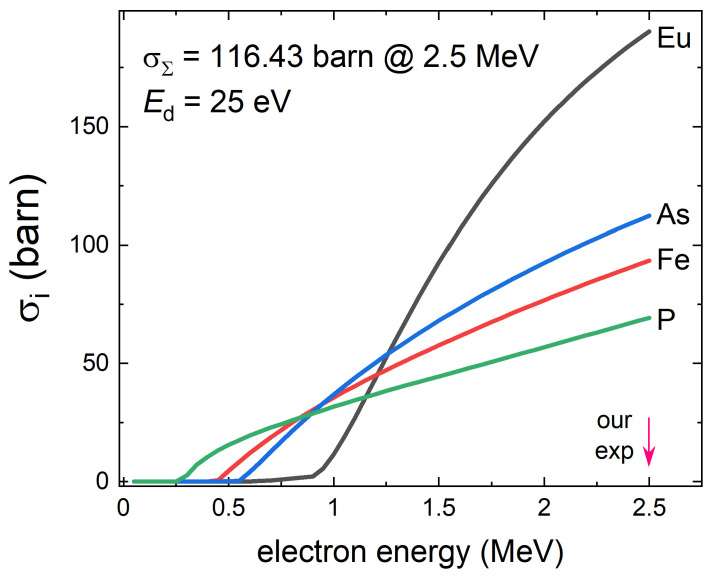
Defects creation cross-sections for different ions in ${\mathrm{EuFe}}_{2}{\left({\mathrm{As}}_{0.77}P_{0.23}\right)}_{2}$ as function of electron energy assuming the displacement energy threshold, $E_{d}=25\;\mathrm{eV}$. At 2.5 MeV, the partial cross-sections are P: 69.2 barn, Fe: 93.5 barn, As: 112.4 barn and Eu: 190.3 barn. The total cross-section of defects creation is estimated as 116.4 barn, which leads to the estimate of the $7.3\times 10^{-4}$ displacements-per-atom (dpa) per 1 C/cm${}^{2}$ of the irradiation.

Figure 3 shows partial cross-sections of the defects creation calculated using SECTE software, developed at Ecole Polytechnique (Palaiseau, France) specifically for electron irradiation.

Of course, the greatest uncertainty is the displacement threshold energy barrier, $E_{d}$, which varies between 10 and 50 eV for different ions and compounds [86,87,88]. In this work its precise value is not important since we only need the order of magnitude estimate. We used a typical value of 25 eV commonly assumed for cross-section calculations for both electron and proton irradiations [86]. This gives around 0.07 at.% dpa (displacements-per-atom) per $1\;C/\mathrm{cm}{}^{2}$ of electron irradiation or about 7 defects-creating collisions per 1000 unit cells (10 atoms in a *Z* = 2 unit cell) and about twice that value for protons. Therefore the density of the defects is small and they do not alter chemical composition and do not “dope” the system, which was proven by Hall effect measurements in another 122 compound, B${}_{1-x}$K${}_{x}$Fe${}_{2}$As${}_{2}$ [78]. Examination of Figure 3 shows that irradiation at our energy of 2.5 MeV produces mostly defects on the Eu sites, whereas lower energy, say 1 MeV, would produce the least defects on the Eu sites. Such energy-tuneable irradiation is possible and would lead to ion-specific study of the effects of disorder.

**Figure 4:**
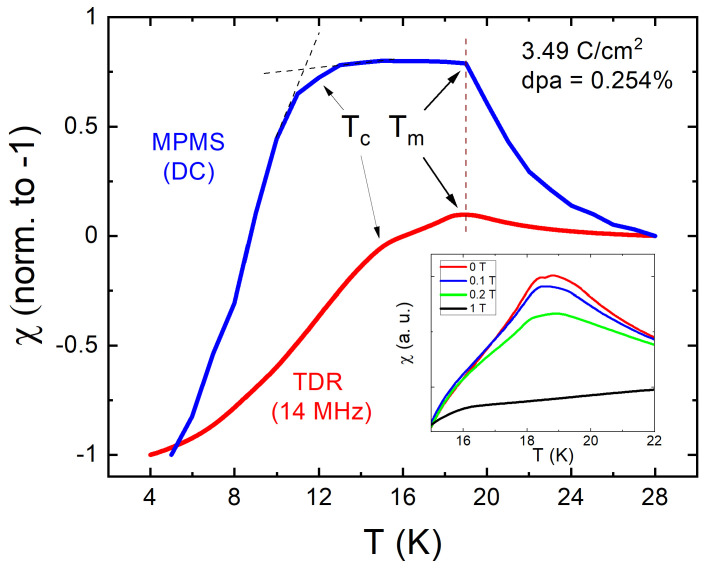
Magnetic susceptibility of ${\mathrm{EuFe}}_{2}{\left({\mathrm{As}}_{0.77}P_{0.23}\right)}_{2}$ single crystal after 2.5 MeV electron irradiation with a dose of 3.49 C/cm${}^{2}$ (0.254 at.% dpa) measured in a DC regime using MPMS (top blue curve) and at 14 MHz using TDR (red curve). The inset shows the evolution of the TDR peak near the ferromagnetic transition for different applied magnetic fields.

It is important to note that we studied physically the same crystals before and after the irradiation, so the observed changes are the results of the added disorder. Figure 4 shows the temperature dependent susceptibility of electron irradiated sample with the dose of 3.49 C/cm${}^{2}$ measured using MPMS (blue curve) and TDR (red curve). Both measurements clearly show a very significant $T_{c}$ suppression, but only a modest increase of $T_{m}$. This leads to an outstanding result that the irradiation has driven the superconducting transition from well above $T_{m}$ to well below. Therefore, we have a unique situation that both regimes could be studied in the same sample. One of the important properties is the transition temperature itself as the function of disorder. While in the regime of $T_{c}>T_{m}$ both measurements gave similar $T_{c}$, see Figure 1, we see very a different $T_{c}$ in the irradiated sample measured by the two techniques when $T_{c}<T_{m}$. Clearly, the difference is due to the dynamic nature of the superconducting transition. Now the superconductivity develops on a ferromagnetic background, hence in the presence of a finite internal magnetic field and, therefore, the nature of the transition is reminiscent of the magnetic irreversibility temperature, which is known to be very frequency-dependent [89] in materials with large magnetic relaxation, such as high$-T_{c}$ cuprates [90,91] and iron pnictides [92,93,94].

**Figure 5:**
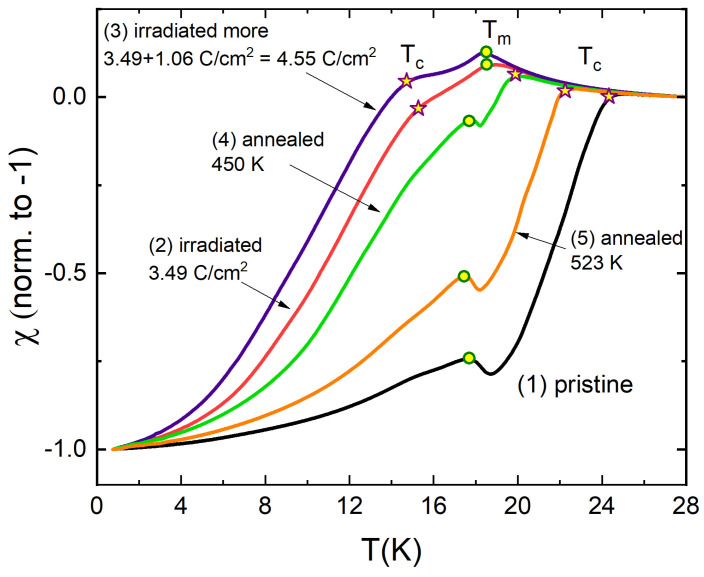
Electron irradiation and subsequent annealing studies of the same ${\mathrm{EuFe}}_{2}{\left({\mathrm{As}}_{0.77}P_{0.23}\right)}_{2}$ sample as shown in Figure 4. Curve (1): pristine state; (2): after 3.49 C/cm${}^{2}$ irradiation; (3) 4.55 C/cm${}^{2}$ total dose where 1.06 C/cm${}^{2}$ was added after step (2); (4) after annealing at 450 K, and (5) after the second annealing at 523 K. Stars mark the superconducting transition and the circles mark the ferromagnetic transition.

In the previous studies, we found that defects introduced by the electron irradiation can be annealed leading to the recovery towards the pristine state, sometimes almost completely [95,96]. Figure 5 shows the evolution of the dynamic magnetic susceptibility measured using TDR first after two subsequent irradiation runs and then after two steps of annealing. Curve (1) shows the pristine state; (2): after 3.49 C/cm${}^{2}$ irradiation; (3) at 4.55 C/cm${}^{2}$ total dose where 1.06 C/cm${}^{2}$ was added after the preceding step; (4) after annealing at 450 K, and (5) after the second annealing at 523 K. The annealing was done in argon atmosphere for several hours and then cooling overnight before opening the chamber. We observe a remarkable practically reversible transformation from the initial state with $T_{c}>T_{m}$ to the state with $T_{c}<T_{m}$ and back to $T_{c}>T_{m}$ again. The stars mark the apparent superconducting transition and the circles mark the ferromagnetic transition. Therefore, the superconducting state can be switched off by the electron irradiation and recovered by the annealing, leaving magnetism practically intact, thanks to the local nature of Eu moments. It is quite different in the case of itinerant magnetism of iron where the magnetic transition is suppressed at the same large rate as the superconducting transition [77]. Here, $T_{m}$ slightly increases by less than a degree when magnetism sets in in the normal metal. This shows that superconductivity weakens (screens) the exchange interaction suggesting, although indirectly, that two phases coexist microscopically.

**Figure 6:**
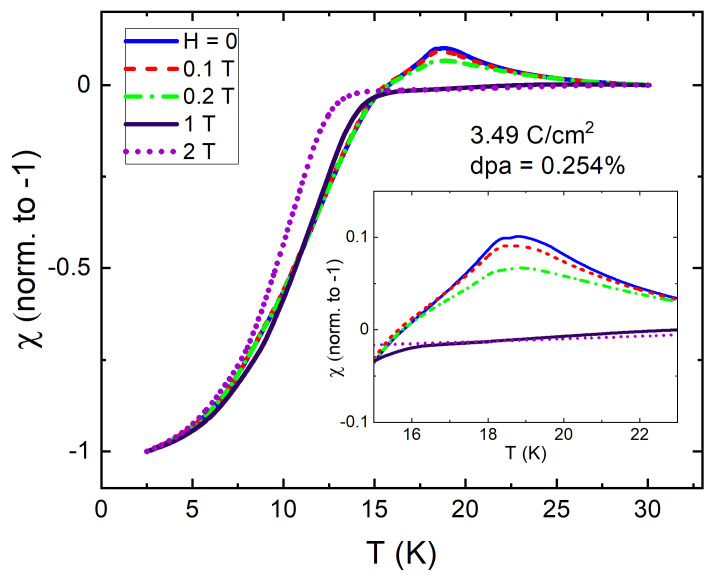
The magnetic-field dependence of TDR dynamic magnetic susceptibility in a 3.49 C/cm${}^{2}$ electron irradiated sample. The indicated magnetic fields were applied parallel to c-axis. The inset zooms on the transition region.

When studying superconductors, it is often needed to reveal the behavior of the normal state “behind” the superconducting response. For example, to estimate the phonon contribution to the specific heat. A common recipe is to apply strong enough magnetic field and suppress superconductivity. However, in ferromagnetic superconductors, with relatively high $T_{m}$ and $T_{c}$, the specific heat jump at the superconducting transition temperature, is hardly detected/resolved, since the magnetic contribution to the specific heat can be large [27,97]. Therefore, our results provide an alternative method to reveal the normal state and, if needed, recover back the superconducting state by annealing. Furthermore, this way of $T_{c}$ suppression by the irradiation without altering chemical composition can be applied for quantitative specific heat studies of other ferromagnetic superconductors even with $T_{c}\leq T_{m}$, in order to deduce magnetic and superconducting volume fractions by moving $T_{c}$ further down to show that both FM and SC phases are bulk in nature (or not). This also allows studying the influence of moderate magnetic fields on the FM transition that is linked to the character of magnetism [85]. By suppressing the superconducting state by electron irradiation we reveal the local nature of ferromagnetism in ${\mathrm{EuFe}}_{2}{\left({\mathrm{As}}_{0.77}P_{0.23}\right)}_{2}$. This follows from the behavior of the peak in dynamic susceptibility in the normal state. Figure 6 shows the evolution of a ferromagnetic peak with the applied DC magnetic field along c-axis. Upon cooling from above $T_{m}$, TDR measurements in local-moment systems exhibit a sharp peak in zero field. When a small magnetic field is applied, it reduces the amplitude of the peak as shown in the inset in, Figure 6. In case of itinerant ferromagnetism there is broad maximum rapidly smearing and shifting to the lower temperatures [85].

**Figure 7:**
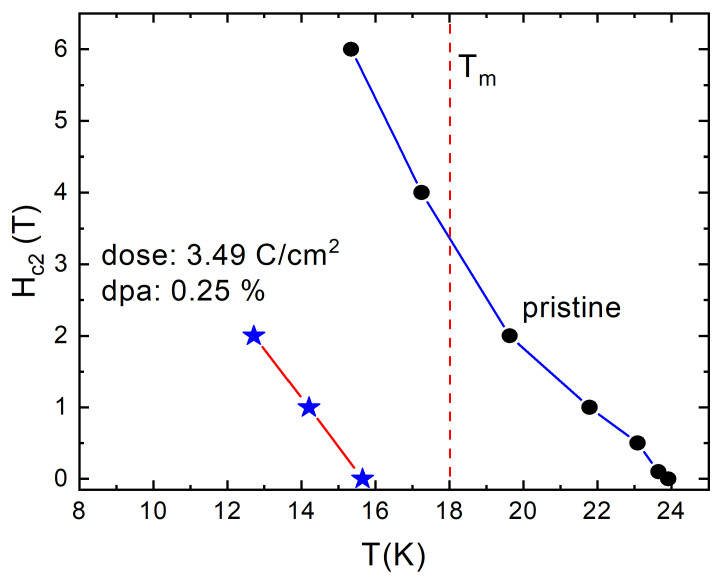
Upper critical field, $H_{c2}\left(T\right)$, of pristine (black circles) and 3.49 C/cm${}^{2}$ electron irradiated (blue stars) ${\mathrm{EuFe}}_{2}{\left({\mathrm{As}}_{0.77}P_{0.23}\right)}_{2}$ with the magnetic field applied along the *c*-axis. Note that the slope, $dH_{c2}\left(T\right)/dT$ near $T_{c}$ in irradiated state is larger than the slope in the pristine state.

Finally, we compare the upper critical field, $H_{c2}\left(T\right)$, in pristine state (black filled circles in Figure 7) and in a state after after 3.49 C/cm${}^{2}$ electron irradiation (blue stars) of the same sample. The data for the pristine sample are close to the values reported for a polycrystalline sample [97]. While it is expected that $H_{c2}\left(T\right)$ may have a step-like feature at $T_{m},$ we did not have an opportunity to study the $H_{c2}\left(T\right)$ line in great detail and it is impossible to draw any conclusions from our data. Yet, the curve shows an unusual positive curvature entering the region of $T\leq T_{m}$, which is not expected in standard models [98]. We can speculate that the magnetic pair-breaking scattering is reduced in the long-range ordered phase, because it requires spin-flip of the scatterer. This will cause an increase of $H_{c2}\left(0\right)$ [47].

Furthermore, the slope, $dH_{c2}\left(T\right)/dT$ near $T_{c}$ is proportional to $T_{c}$ multiplied by a function of potential and pair-breaking scattering [47]. That function increases with the increase of the potential scattering and decreases with the increase of the pair-breaking one. According to the Anderson theorem, potential scattering does not change $T_{c}$ whereas pair-breaking scattering decreases $T_{c}$. Note that in sign-changing $s_{\pm }$ order parameter, the interband potential (non spin-flip) scattering is also pair-breaking, while the inband potential scattering is not, provided that each band has no nodes or significant anisotropy [99]. Figure 7 shows that the slope at lower $T_{c}$ is actually larger than that in the larger $T_{c}$ pristine state indicating that the pair-breaking scattering increases $H_{c2}$ faster than it suppresses $T_{c}$ adding to the conclusion that electron irradiation produces a substantial amount of the additional pair-breaking scattering.

### 3.2. Phase Diagram and Comparisons with Other Compounds and Irradiation

The response to any perturbation, irradiation included, should be gauged against the results obtained with other types of materials and irradiations. Here we compare the results with CPWR measurements of proton-irradiated samples. Protons also introduce largely point like defects and, in addition, nanometric clusters, which slightly reduce the efficiency of the overall produced defects acting as scattering centers. Detailed account of the effects of disorder by doping and proton irradiation in ${\mathrm{EuFe}}_{2}{\left({\mathrm{As}}_{0.77}P_{0.23}\right)}_{2}$ is given elsewhere [66].

**Figure 8:**
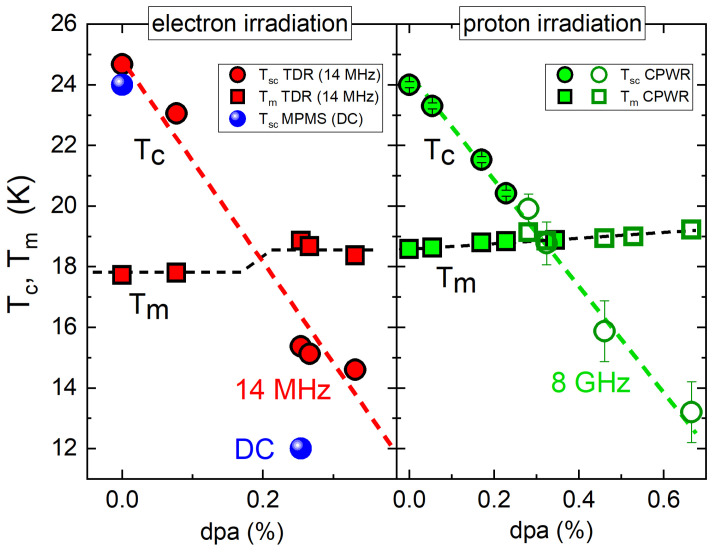
Transition temperatures vs. the estimated atomic concentration of defects in ${\mathrm{EuFe}}_{2}{\left({\mathrm{As}}_{1-x}P_{x}\right)}_{2}$ single crystals obtained by different techniques and different doses of irradiation. (Left panel) electron irradiation in *x* = 0.23 crystal, measurements using (red) tunnel-diode resonator (TDR) and (blue) DC magnetometry; (Right panel) proton irradiation in *x* = 0.23 (hollow symbols) and *x* = 0.20 (full symbols) crystals, measurements using coplanar waveguide resoantor (CPWR). The way the effective disorder has been estimated in this case was discussed in [66].

Figure 8 shows the superconducting and ferromagnetic transition temperatures versus the estimated atomic percentage of induced defects. The displacements-per-atom (dpa) values are based on the calculated cross-sections using SECTE for electron irradiation and SRIM for proton irradiation [100]. We therefore present the data on two panels, since the dpa values are obtained using different calculations. Regardless of the type of irradiation or experimental time window, the magnetic transition remains robust and stays around $T_{m}\approx$ 18 K only slightly increasing in the normal state. The superconducting transition, while suppressed significantly, decreases at a similar rate for different experimental methods operating at very different frequencies as long as $T_{c}>T_{m}$. However, the agreement breaks after the transition temperatures swap places, $T_{c}<T_{m}$. There are two major contributions at play here. One is the time window of the measurement, which leads to lower $T_{c}$ for smaller frequencies (longer relaxation). The second parameter is the nature of the defects, considering that electrons and protons have not only very different masses, but also opposite signs of their charge. Unfortunately, we do not have ion-type resolved particle cross-sections for the proton irradiation, but it is quite possible that protons knock out particular ions at rates different from electrons. Here we can conclude that the background magnetism affects superconductivity in a way consistent with the conclusions of the previous studies - the superconducting phase develops with the magnetic field present, which immediately triggers significant time-dependencies of all measurable parameters.

**Figure 9:**
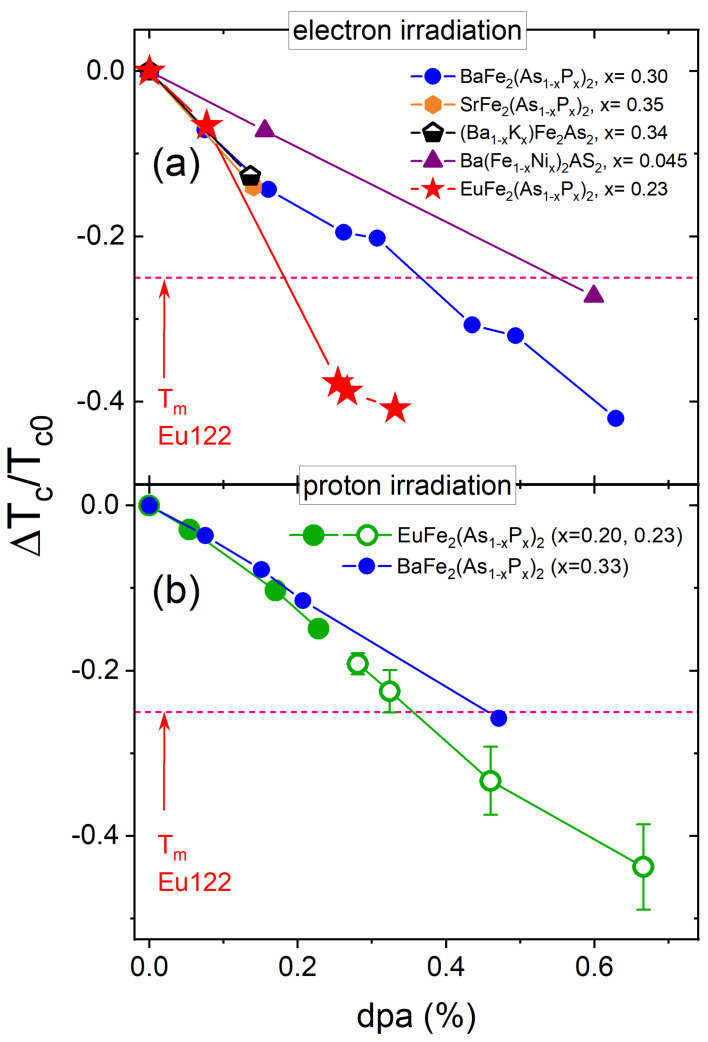
The normalized rate of $T_{c}$ suppression by electron irradiation (panel (**a**)) and proton irradiation (panel (**b**)). Electron irradiation results from this work are compared to the data in other compounds, summarized in Ref. [59]. The data for the important for direct comparison ${\mathrm{BaFe}}_{2}{\left({\mathrm{As}}_{1-x}P_{x}\right)}_{2}$ are from Ref. [63]. The dpa percentages were calculated for each of the listed compounds using SECTE calculations similar to Figure 3. For CPWR data, hollow symbols were obtained from a crystal with additional doping-induced disorder (*x* = 0.23). The way the effective disorder has been estimated and its data merged to that of the *x* = 0.20 was discussed in [66].

Figure 9 compares the normalized rate of the $T_{c}$ suppression, $\Delta T_{c}/T_{c0}$, plotted versus the estimated density of the induced defects, which was calculated for each of the listed compounds. Similar to Figure 9, two panels show the results for electrons and protons, respectively. Panel (a) summarizes the results of electron irradiation, while panel (b) shows proton irradiation. We stress that the dpa values are estimated using two different approaches and, also, do not take into account recombination upon warming and possible clusterization and agglomeration into larger non-point like groups. Further controlled studies on similar samples are needed to compare electron and proton irradiation in a quantitative way. Here we see that the suppression rate for electron-irradiated ${\mathrm{EuFe}}_{2}{\left({\mathrm{As}}_{0.77}P_{0.23}\right)}_{2}$ is higher compared to others, non-magnetic, compounds of the IBS. Most likely this is because of the formation of magnetic scattering centers on Eu sites, in addition to the non magnetic channel formed by all defects. Considering partial cross-sections shown in Figure 3 this scenario is quite plausible. In the case of ${\mathrm{BaFe}}_{2}{\left({\mathrm{As}}_{1-x}P_{x}\right)}_{2}$ irradiated by 3 MeV protons, we also found that the suppression rate of $T_{c}$ is larger compared with similar compounds. We attribute this enhanced suppression of $T_{c}$ to the generation of Fe${}_{2}$P, which is one of possible magnetic compounds that can also generate magnetic scattering [101].

## 4. Conclusions

In summary, we used controlled disorder produced by electron and proton irradiations to induce two states in the same sample: (1) $T_{c}>T_{m}$ and (2) $T_{c}<T_{m}$. The ferromagnetic transition, $T_{m}$, is weakly affected by the irradiation whereas the superconducting transition, $T_{c}$, is rapidly suppressed. We therefore had a unique opportunity to study the same ferromagnetism in normal and superconducting background and, vice versa, superconductivity developing in a paramagnetic or a ferromagnetic background. The ferromagnetic transition temperature increases by less than a degree in the normal state compared to when it is born out of superconducting background signaling of the microscopic coexistence and, perhaps, some weakening of the exchange interaction by the superconducting phase. Furthermore, we conclude that in ${\mathrm{EuFe}}_{2}{\left({\mathrm{As}}_{1-x}P_{x}\right)}_{2}$ local-moment ferromagnetism of Eu${}^{2+}$ sublattice does not have a direct impact on superconducting pairing, but it affects the superconducting state via the spontaneous internal magnetic field that creates Abrikosov vortices and antivortices in the neighboring domains. When $T_{c}<T_{m}$, the superconducting transition becomes significantly frequency-dependent reminiscent of the irreversibility temperature, $T_{irr}\left(H\right)$, rather than the true zero-field transition $T_{c}\left(H=0\right)$. It is also possible that the annihilation of vortex-antivortex pairs at the domain boundaries assisted by an AC field at $T_{c}<T_{m}$ can further enhance the frequency dependence due to the dynamic response of shaking-depinned vortex-antivortex lattice, which is different from that of a conventional mixed vortex state. Another effect of Eu${}^{2+}$ sublattice is to provide the effective pair-breaking “magnetic” defects upon particle irradiation. This leads to even faster $T_{c}$ suppression by disorder than in non-magnetic 122 compounds. This also means that the pairing state of ${\mathrm{EuFe}}_{2}{\left({\mathrm{As}}_{1-x}P_{x}\right)}_{2}$ is most likely $s_{\pm }$ as in other IBS.

## Data Availability

The data presented in this study are available on request from the corresponding author.
